# Tunable photoluminescence and energy transfer of Eu^3+^,Ho^3+^-doped Ca_0.05_Y_1.93-x_O_2_ nanophosphors for warm white LEDs applications

**DOI:** 10.1038/s41598-022-09630-x

**Published:** 2022-04-06

**Authors:** Arpita Dwivedi, Monika Srivastava, Amit Srivastava, Chandan Upadhyay, Sanjay Kumar Srivastava

**Affiliations:** 1 Department of Physics, Institute of Science, Banaras Hindu University (BHU), Varanasi, 221005 India; 2 School of Materials Science and Technology, Indian Institute of Technology (BHU), Varanasi, 221005 India; 3 Department of Physics, TDPG College, VBS Purvanchal University, Jaunpur, 222001 India

**Keywords:** Materials for devices, Materials for optics

## Abstract

A series of Eu^3+^ ions doped Ca_0.05_Y_1.93-_xO_3_:0.02Ho^3+^ (CYO:Ho^3+^,xEu^3+^) nanophosphors having multicolour tuneability have been synthesised by following a simplistic solution combustion approach. The synthesised samples have been characterised by employing X-ray diffraction (XRD), Transmission electron microscope (TEM), and Fourier transforms infrared spectroscopy (FTIR). The optical properties have been engrossed by UV–visible and photoluminescent excitation and emission spectra, and decay lifetimes measurements. The characteristic emission, which occurs due to the f-f transition of Ho^3+^ and Eu^3+^ has been observed in emission spectra with excitation of 448 nm. By adjusting the doping ratio of Ho^3+^/Eu^3+^, the as-synthesized nanophosphor accomplishes multicolour tunability from green-yellow to red. Emission spectra and decay lifetime curve recommend dipole–dipole interaction causes energy transfer from Ho^3+^ → Eu^3+^. The energy transfer process from Ho^3+^ to Eu^3+^ has been confirmed through electric dipole–dipole interaction with critical distance 15.146 Å. Moreover, temperature dependent emission spectra show the high thermal stability with an activation energy ⁓ 0.21 eV, with the quantum efficiency of 83.6%. CIE coordinate illustrates that the singly doped Ho^3+^ and Eu^3+^ lie in the green and red region, respectively, while the as-synthesized CYO:Ho^3+^,xEu^3+^shows tunability from green to red with low CCT and high colour purity values. Hence, the CYO:Ho^3+^,xEu^3+^nanophosphor may be a near-UV excited multicolour colour-tunable pertinent candidate with potential prospects for multicolour- display and near-ultraviolet lighting applications.

## Introduction

In recent year, various researches have been reported that the lanthanoid doped nanophosphors are capable to produce multicolor emission with their exhibit distinctive features such as long-lifetime, low cost, high energy saving, higher brightness, fast response, less thermal radiation, and ecological friendly^1–5^. Due to these properties rare earth doped nanophosphor materials are extensively used is numerous applications, such as for optical devices, plasma display devices, flat panel devices, light emitting diodes, and for solid state lighting. Moreover, rare earth doped nanophosphor are also widely used for their application in the different areas such as laser, solar cells, metal ion sensor, bio-imaging, temperature and stress sensor, and radiation damage sensor, etc^6–8^.

Generally, there have been several ways to develop luminescent material with multicolour tunable emission. Some of them are choosing different sensitizes and active ions in a single host, by adjusting the host composition doping with specific active ions, and crystallographic site engineering/doping of rare earth ion at particular host side. Now a days, in order to attain multicolor tunability or white-emitting nanophosphor, energy transfer from sensitizer to an activator is more dynamic area of research^9,10^. Various Energy transfer phosphor materials such as Ca_14_Al_10_Zn_6_O_35_: Bi^3+^, Eu3^+^^9^Ba_9_Lu_2_Si_6_O_24_:Bi ^3+^,Eu ^3+^^5^ ZnMoO_4_:Eu^3+^, Bi^3+^^8^TeO_2_GeO_2_Nb_2_O_5_: Eu^3+^ Ho^3+^^6^and Ca_3_Gd(AlO)_3_ (BO_3_) _4_:Tb^3+^, Eu^3+^^10^Sr_2_Mg_3_P_4_O_15_: Mn^2+^,Eu^2+^^11^ have been studied which display the tunable emission from blue, purple, to red, red to orange/yellow to green and green orang to red, respectively. Hence, Bi^3+^, Tb^3+^, Ho^3+^and Mn^2+^ can be used to sensitize Eu^3+^ ions in host lattice, and in turn multicolor tunablity for plasma display devices, light emitting diodes, solid state lighting and for other applications^6,8–11^. However, these phosphors emit strong red emission with UV excitation but the emission intensity falls to nearly one fifth with excitation by near UV radiation and also illustrate less colour purity with weak thermal stability. Therefore, there is still a craving for the development of new phosphor material and the evolution of existing one to overcome these associated limitations. So far, no reasonably relevant phosphor has been found which may be considered as an effective approach to realize the multicolour tunable emission.

Moreover, among the rare-earth ions, Ho^3+^ and Eu^3+^ act as an excellent activator due to their effective green (^5^S_2_,^5^F_4_) → ^5^I_8_ (Ho^3+^) and red ^5^D_0_ → ^7^F_2_ (Eu^3+^) emission, respectively, to realize multicolour emission^6,12,13^. The trivalent holmium (Ho^3+^) doped phosphor displays a wide luminescence spectrum from the blue to IR region. The energy levels of Ho^3+^has a 4f.-electronic configuration and thus allow intra-4f. transitions which exhibit strong green emission attributing to the ^5^S_2_,^5^F_4_ → ^5^I_8_ transition with weak red (^5^F_5_ → ^5^I_8_) emission in various hosts^12,14^. Due to weak emission in the red region, the colour rendering index of singly doped Ho^3+^ is low. Thus, the generation of white light with Ho^3+^ activator is possible, if this deficiency could be overcome by doping with red-emitting phosphor. So, the trivalent Eu^3+^ ions can be used as a dopant, as Eu^3+^ has intense red–orange emission corresponding to ^5^D_0_ → ^7^F_j_ (*j* = 1,2,3,4,) transitions^13,15^. However, commercially available red phosphors have low efficiency, are chemically unstable and usually decompose at higher temperatures^13,15^. In this context, to identify a suitable host material Yttrium oxide (Y_2_O_3_) has been studied which suggests it to be one of the most appropriate hosts as it can easily be doped with various rare-earth ions owning to comparable chemical and ionic radii. Additionally, Y_2_O_3_ exhibits excellent physical properties such as high melting point, low thermal expansion. Furthermore, the wide optical band gap (5.8 eV) of the host decreases the effect of optical absorption and the small phonon energy increases the probability of radiative transitions with easy and low synthesis cost etc.^14,16^. The doping with Ca^2+^ fringes to reduce the sintering temperature, generates asymmetry in lattice, and also produces oxygen vacancies which assist fast energy transfer between host to Eu^3+^ions^17–22^. This can significantly enhance the PL emission intensity, and Ca_0.05_Y_1.93-x_O_3_ (CYO) can act as a substantial host for synthesis^23,24^. Despite these peculiar features, the substantial Eu^3+^-doped Ca_0.05_Y_1.93-x_O_3_: Ho^3+^ (CYO:Ho^3+^,xEu^3+^) phosphor has not been studied widely so far. In the present work, foremost we have successfully synthesized CYO:Ho^3+^,xEu^3+^nanophosphor by a simple solution combustion process and subsequently examined its tuneable multicolour luminescence properties. Under near-UV (448 nm) excitation, as-synthesized nanophosphor illustrates an intense emission in the visible region due to the energy transfer from Ho^3+^-Eu^3+^. The colour tunability of Ho^3+^and Eu^3+^ doped CYO nanophosphor from green to red depends on the Eu^3+^ concentration. These findings establish that CYO:Ho^3+^,xEu^3+^multicolour emitting nanophosphor as a promising entrant to be used as multicolor tuneable for optoelectronic and display applications.

## Results and discussion

### XRD patterns, Phase structure and Rietveld refinement

**Figure 1:**
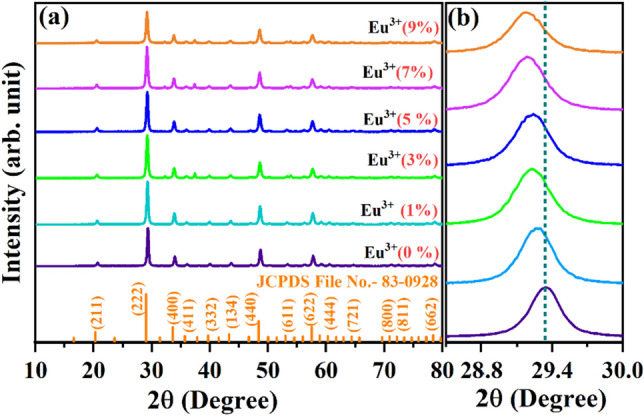
(**a**) X-ray diffraction pattern of CYO:Ho^3+^, xEu^3+^ where x = 0, 1, 3, 5, 7, 9 mol% (**b**) magnified view of the diffraction peak (222) showing shift in 2θ toward higher angle side due to incorporation of Eu^3+^ with Ho^3+^ in the lattice of CYO.

Figure 1a depicts the XRD patterns of CYO:Ho^3+^,xEu^3+^ (where x = 0, 1, 3, 5, 7, 9 mol%) nanophosphors in the diffraction 2θ range 10°-80°. The major diffraction peaks at (20.4°, 29.1°, 33.7°, 48.5°, 57.4°) correspond to the reflection planes (211), (222), (400), (440), (622), accompanied by some minor peaks (39.8°, 43.4°, 53.1°, 60.4°, 64.4°, 71.0°, 72.3°) matches well with (332), (134), (611), (444), (721), (800) and (811) reflection planes (JCPDS card No.: 83–0928) of Y_2_O_3_. The appearance of intense and broad peaks specifies that the particles of the as-synthesized nanophosphors to be well crystallized and nanometre range ^25,26^. These findings indicate that the phase of as-synthesized CYO:Ho^3+^,xEu^3+^ nanophosphors are pure i.e. there have been no traces of unreacted constituents or impurities. The coordination number of Y_2_O_3_ is six and has a cubic bixbyite structure. Yttria unit cell contains a total 32 sites, 24 at C_2_ sites and 8 at C_3i_ or S_6_ sites, which is substituted during doping. The comparable ionic radii of Ho^3+^ (0.901 Å), Eu^3+^ (0.947 Å) and Y^3+^ (0.900 Å) and have allowed the successful replacement of Y^3+^ ions by the dopants in the CYO host, and this substation also favoured by charge balancing state^27,28^. There are also a slight shift observed in XRD peak towards the lower angle side after substitution of Ho^3+^/Eu^3+^ in CYE.

Figure 1b represents the magnified view of the most intense peak corresponding to the reflection plane (222). It signifies that the amount of % mole doping of Eu^3+^ influences the peak intensity, it decreases with the doping concentration and also leads to a small shift towards the lower angle side at higher concentration. This finding demonstrates that Ho^3+^ and Eu^3+^ ions have been successfully doped in CYO:Ho^3+^,xEu^3+^ samples. Malek et. al. also mentioned a similar type of shifting with doping of Eu^3+^ in Y_2_O_3_^13^. Due to consequence, the estimated value of lattice constant (a) of host lattice increases from 10.61 (Eu^3+^ 0%) to 10.614 (Eu^3+^ 5%), and cause lattice expansion. The crystallite size of the nanophosphor has been estimated using the most instance diffraction peak (222) through Debye Scherrer’s formula given in (1) ^29^:

1 $$D = \frac{0.89\lambda }{{\beta \cos \theta }}$$

where D represents the crystallite size, K is a constant (K⁓ 0.89), λ refers to the wavelength of the x-rays used (0.15405 nm), θ corresponds to the angle of diffraction, whereas β is the full width at half maximum (FWHM in radian). The calculated values of crystallite size lie in the 22–27 nm range. Similar trends have been reported by Ramgopal et al.^30^. It has been observed that the β, full width at half maximum (FWHM in radian) of the XRD diffraction peak may influence both the lattice strain and crystallite size which can easily be understood by Williamson–Hall equation as given by Eq. (2)^31^

2 $$\beta Cos\left(\theta \right)=\frac{K\lambda }{D}+4\varepsilon Sin(\theta )$$

**Figure 2:**
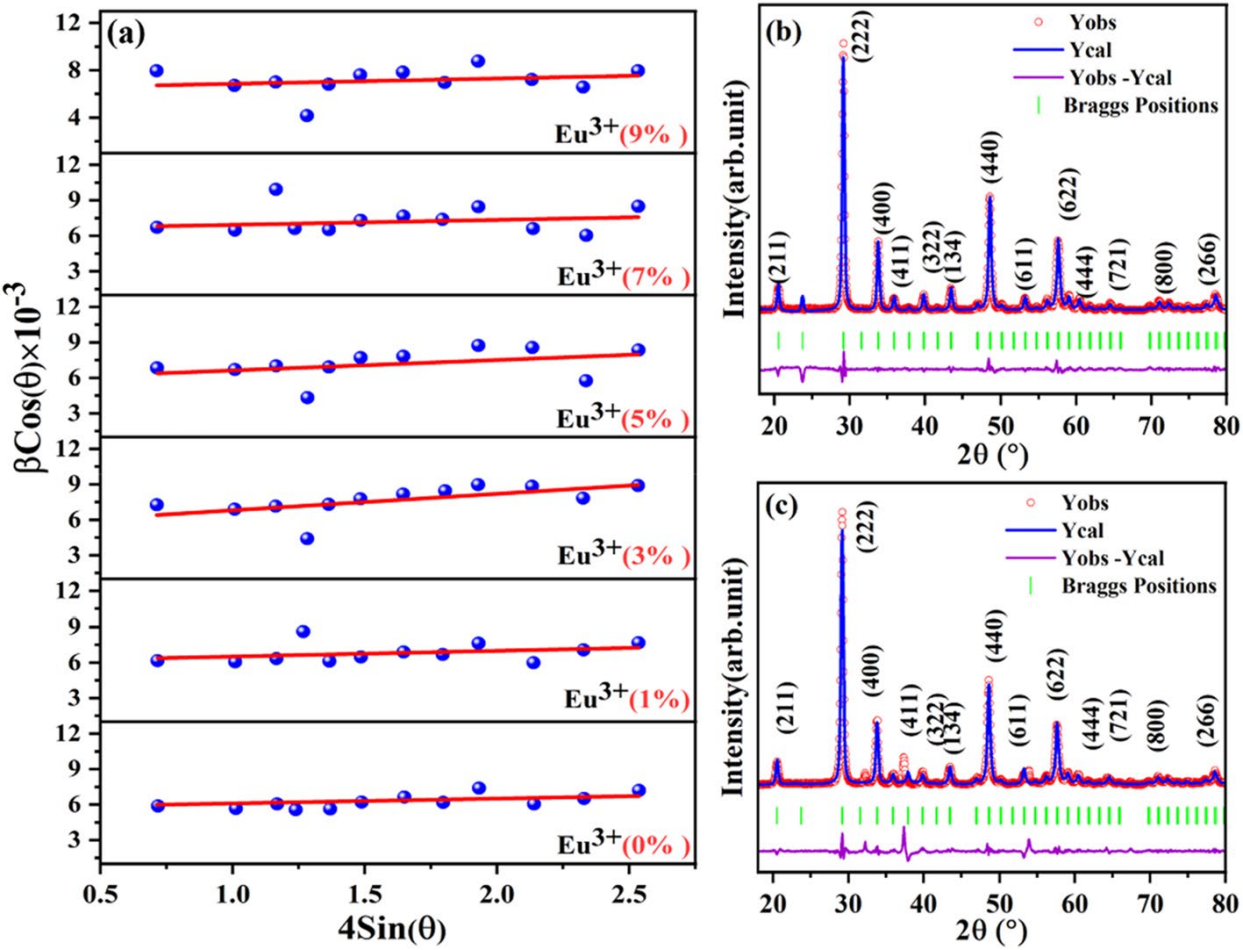
(**a**) W–H plot of CYO:Ho^3+^,xEu^3+^ nanophosphors from x = 0, 1, 3, 5, 7, 9 mol% (**b**) Rietveld refinement of XRD pattern x = 0 mol% and (**c**) x = 5 mol% of CYO:Ho^3+^,xEu^3+^ samples respectively.

The lattice strain (ε) has been estimated from the slope of the βcos (θ) versus 4sin (θ) W–H plot and the crystallite size from the intercept of K/Dλ on the y-axis. The crystallite size has been found to repose in the 21–25 nm range and further, the estimated values from the W–H plot have been summarized in Table 1 and the corresponding plot has been presented in Fig. 2a. The crystallite size (D) and effect of strain on CYO:Ho^3+^,xEu^3+^ samples have been determined by using both Debye Scherrer’s formula and Williamson–Hall (W–H) plots. There has been little variation in crystallite size as estimated by Scherrer’s, and W–H plots. This is because the strain component in Scherrer’s equations is usually assumed to be zero. The W–H plot shows that with the increase in Eu^3+^ ions concentration, the micro strain also increases and consequently causes a shift in the peak position towards lower angle side. Therefore, the substitution of Eu^3+^ with CYO:Ho^3+^ lattice leads to the distortion of periodicity of the host lattice. This has also been approved by the increase in the lattice constant of CYO:Ho^3+^,xEu^3+^ as compared to the CYO:Ho^3+^ nanophosphor. Furthermore, higher microstrain values might cause a decrease in the fluorescence intensity. In the present case, the estimated strain present in the as- synthesised nanophosphors are very small which lead to a negligible effect on the PL- intensity of the doped samples even at higher concentration of Eu^3+^ ions. One other structural parameter; dislocation density ($\delta =\frac{1}{{D}^{2}}$; where, δ represents the dislocation density and D as crystallite size) has also been evaluated and put in Table 1^30^. The low values of dislocation density produce large disordered in the crystal, which supports the increment of fluorescence intensity with doping.

**Table 1:** Estimated average crystallite size and lattice strain in the as-synthesized samples through Debye Scherrer’s formula and W–H equation.

Nanophosphor CYO:Ho^3+^,xEu^3+^ (at. mole%)	Crystallite size D_hkl_ (nm)		Microstrain ε (× 10^–4^)	Dislocation density (nm^-2^) from Scherrer formula δ(× 10^–4^)
	Scherrer’s formula	W–H plot		
0	22	22	4.08	20.85
1	22	24	4.18	20.66
3	25	26	4.47	16.52
5	25	26	4.84	15.87
7	26	28	8.80	14.79
9	27	28	13.9	14.13

The FULLPROF suit program has been used for Rietveld refinement analysis of CYO:Ho^3+^,xEu^3+^ (0 and 5% Eu^3+^). Pseudoviogt function has been used to fit various parameters of the data point^29,30^. Figure 2a,b displays the Rietveld refinement of XRD pattern of CYO:Ho^3+^,xEu^3+^ nanophosphor samples with x = 0 mol% and x = 5 mol%, respectively. Cubic structure of Y_2_O_3_ has been considered as an initial reference for the refinement of data to approach the actual crystal structure. In the refinement diagram, the solid line shows the calculated XRD pattern, dots represent the observed XRD patterns, the vertical bars represent the Bragg’s positions of cubic phase and the lower profile show the difference between observed and calculated XRD patterns. The quality of refine data was predicted by lower values of goodness of fit ((GOF) = χ^2^ = (R_p_/R_wp_)^2^) for the best refinement results GOF must approach to unity. The GOF of CYO:Ho^3+^,xEu^3+^ was found to be 13.51 (0 mol%) and 8.01(5 mol%), which confirms good agreements between experimental and theoretical plots. The estimated value of lattice parameter was a = b = c = 10.6081 Å, α = β = γ = 90° and a = b = c = 10.6108 Å, α = β = γ = 90° with unit cell volume 1193.74 Å^3^and 1194.69Å^3^ for 0 mol% and 5 mol% of CYO:Ho^3+^,xEu^3+^ respectively. Refinement also reveals the phase of the nanophosphor was cubic with Ia $\overline{3 }$ (230) space group. Shi, Hui, et al. has also reported the cubic phase after doping Ca^2+^, and Eu^3+^ with Y_2_O_3_^17^. In general, the cubic structure of Y_2_O_3_ has two sites S_6_/C_3i_ (8b) and C_2_ (24d) sites**)**. These sites describe the optical and physical properties of nanophosphor, the S_6_/C_3i_ has inversion centre and only allow magnetic dipole transition. Whereas, C_2_ sites does not has inversion centre, hence allow both electric and magnetic dipole transition and responsible for PL emission intensities^32^. Hence, in CYO:Ho^3+^, xEu^3+^   lattice Y^3+^ (0.900 Å) ions are replaced by Eu^3+^/Ho^3+^ (Eu^3+^ -0.947 Å, Ho^3+^-0.901 Å), and Ca^2+^(1.1 Å) substituted to C_2_ sites as they have comparable radii and responsible for luminescence emission.

### Transmission electron microscopy (TEM)

**Figure 3:**
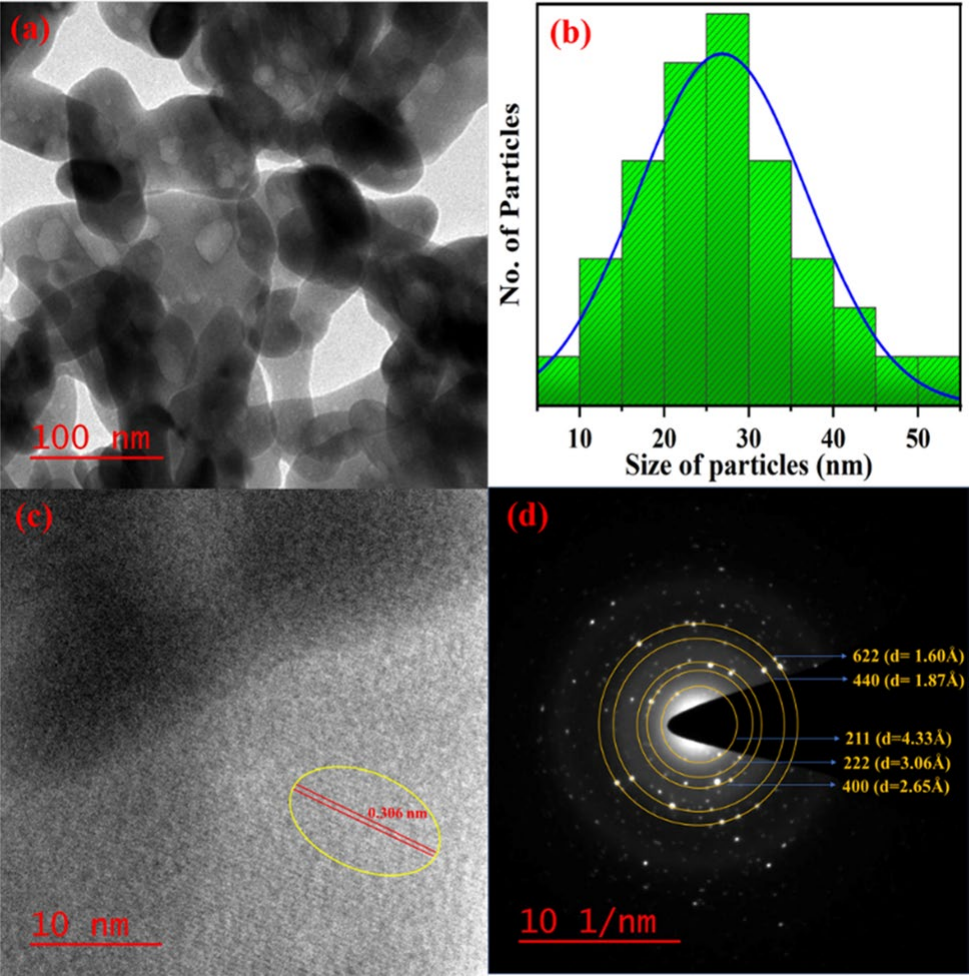
(**a**) TEM image (**b**) particle distributions histogram (**c**) lattice fringes (**d**) SAED analysis of CYO:Ho^3+^,xEu^3+^ (5 mol%) nanophosphor.

To get the particularized information of the homogeneity distribution and to understand the quality of crystal formed, high-resolution transmission electron microscopy (HTEM) and selected area diffraction (SAED) pattern have been carried out and presented in Fig. 3. TEM micrograph of the as-synthesized sample confirms that there have been a large number of small particles associated with each other i.e., particles are highly aggregated (Fig. 3a). The morphology of the sample has not been in uniform size and most of these nanoparticles are agglomerated in spherical, and cubical forms. The nonuniform distribution of spherical, and cubical shaped particles may be due to the non-uniform temperature during the combustion process^12^. The HRTEM image (Fig. 2c), gives evidence that the nanophosphor contains lattice fringes that are clear and reflects the plane (222) with interspacing in 0.306 nm. The average particle size (Fig. 3b), of the sample, has been found as ⁓27 nm which is greater than the crystallite size as obtained from the XRD results, which confirms the agglomeration of the nanophosphor. The SAED pattern indicates the high crystalline nature of CYO:Ho^3+^,xEu^3+^ and indexed well with (211), (222), (400), (440), (622) reflection planes. In the SAED pattern (Fig. 3d), multiple diffraction spots ordered in ring form are present which correspond to high nano crystallinity with the polycrystalline nature of the nanophosphor sample. The TEM and SAED images confirm that as-synthesised nanophosphor exhibits a highly crystalline and less disordered nature. This nature of CYO:Ho^3+^,xEu^3+^ nanophosphor samples assist the strong fluorescence intensity. All these analyses confirm that the Eu^3+^ and Ho^3+^ have effectively been doped into CYO lattices^30,33^.

### Fourier transform infrared spectroscopy (FTIR) analysis

**Figure 4:**
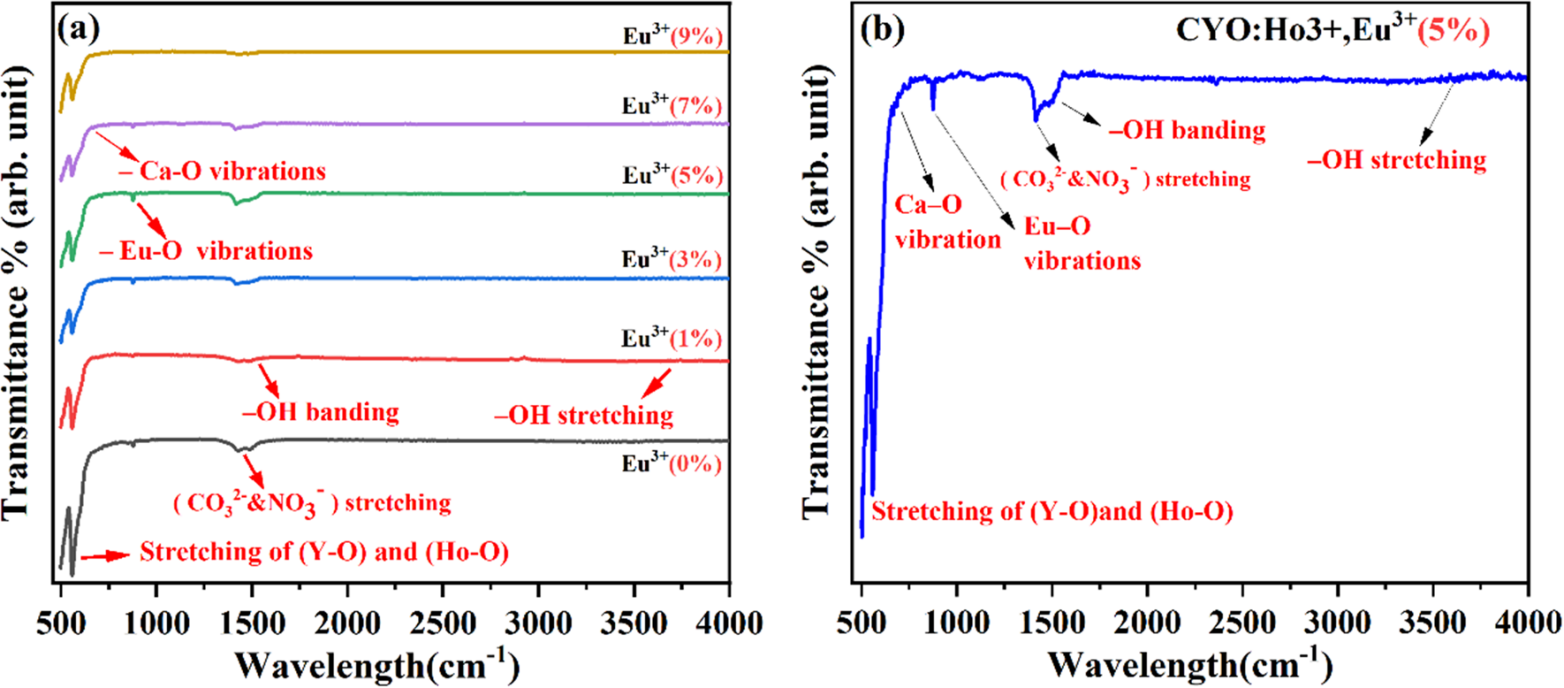
(**a**) FTIR spectra of CYO:Ho^3+^,xEu^3+^ nanophosphors for x = 0, 1, 3, 5, 7, 9 mol% (**b**) FTIR of 5% doped CYO:Ho^3+^,xEu^3+^ nanophosphors with characteristic vibrations.

Figure 4 represents the FTIR spectra of CYO:Ho^3+^, xEu^3+^ nanophosphors for x = 0, 1, 3, 5, 7, 9 mol%. The spectra have been recorded in the range 500–3600 cm^-1^. The emergence of a strong vibrational band near ~ 558 cm^-1^ and weak vibration at ~ 513 cm^-1^ attribute to stretching vibrations of metal–oxygen (Y–O) and (Ho-O) and (Y–O), respectivly^34–36^. The characteristic vibration at 686 cm^−1^ owes to Eu-O^37,38^ and vibrational bands in the range 692–699 cm^−1^ correspond to Ca–O vibrations^39^. Besides, other bands such as bands around at 1425 cm^-1^ and 1515 cm^-1^ are asymmetric stretching vibrations of C-O and band at ~ 1046 cm^-1^ corresponds to the bending vibration of C-O. These C-O bands arise due to the absorption of CO_2_ from the ambient atmosphere on the surface of samples or due to residual carbon during the sample preparations^13^. The absorption band centred at ⁓1391 cm^−1^ is due to the symmetric stretch vibrations of the residual nitro group (NO_3_^-^)^40^. While the weak absorption band at ⁓ 1545 cm^−1^ and ⁓ 3600 cm^−1^ have been due to symmetric stretching vibrations of the hydroxyl group (-OH), This might be due to the absorption of moisture from the atmosphere on the nanophosphor surface. This result indicates that nanophosphor is almost crystalline and decomposed completely with no impurity phase present in the as-synthesised samples.

### UV–Visible absorption studies

**Figure 5:**
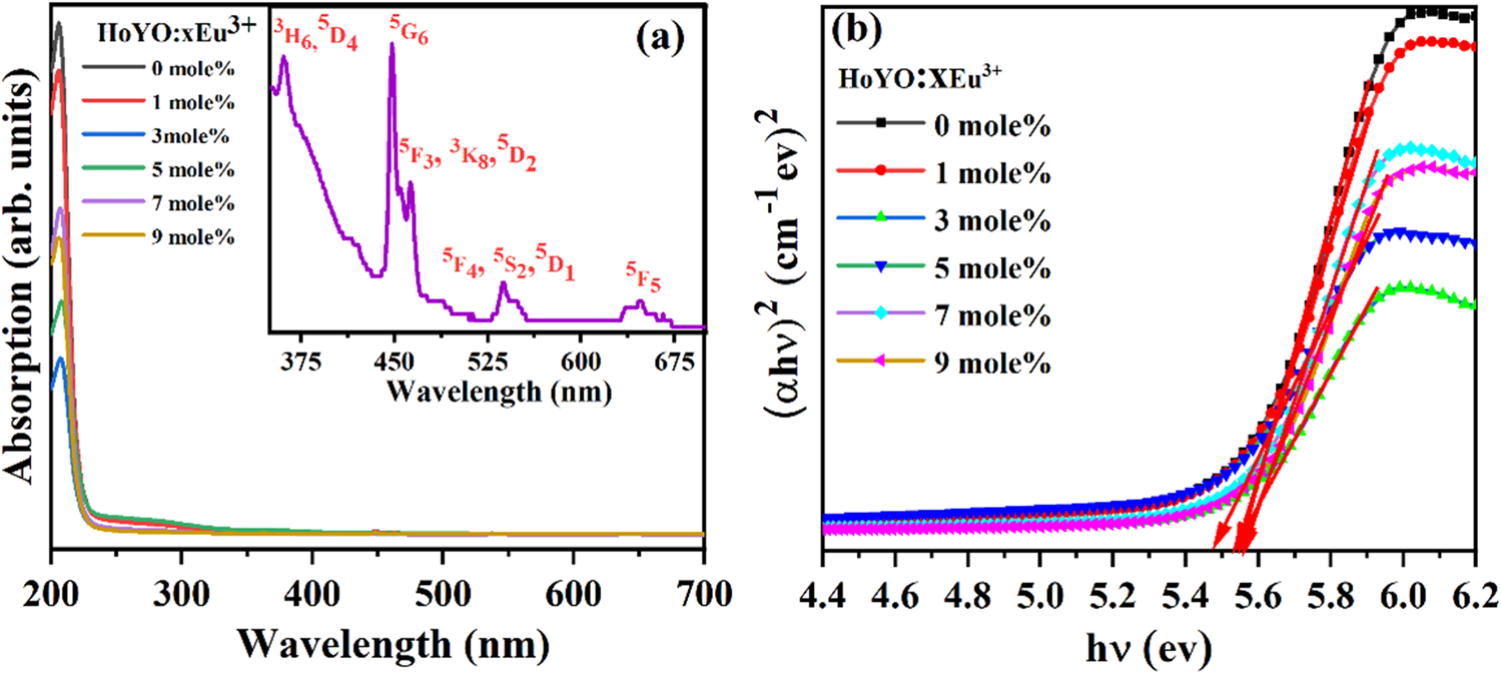
(**a**) UV–Vis absorption spectra of CYO:Ho^3+^,xEu^3+^ (x = 0, 1, 3, 5, 7, 9 mol%) and inset show the enlarged view of CYO:Ho^3+^,5.0Eu^3+^ for 350–700 nm and (**b**) optical energy band gap of CYO:Ho^3+^,xEu^3+^ (0–9% mole) nanophosphor.

The UV–Vis absorption spectra of CYO:Ho^3+^,xEu^3+^ doped with different concentrations of Eu^3+^ (0, 1, 3, 5, 7, 9 mol%) has been illustrated in Fig. 5a. It reveals presence of a sharp peak at ~ 207 nm mainly attributed to the bandgap of CYO:Ho^3+^, xEu^3+30^. This characteristic peak has been appeared due to charge transfer from the valance band of O^2-^ (2p electrons) to the conduction band of Y^3+^ (4d level) corresponding to host lattice. There is also a less intense sharp peak present in the 350–700 nm range which corresponds to the f-f transition of Eu^3+^ and Ho^3+^ as given in the inset of Fig. 5a. The bands at ~ 362, 448, 463, 539, 648 nm owing to the transition of ground state ^5^I_8_ of Ho^3+^ to different upper levels corresponding to ^3^H_6_, ^5^G_6_, ^3^K_8_, ^5^F_3_, ^5^F_5,_ respectively^14^. Besides, bands near at ~ 361 nm, 464 nm, 534 nm correspond to the transition from ^7^F_0_ to ^5^D_4_, ^5^D_2_, ^5^D_1_, in Eu^3+^ ions, respectively^34^.

The optical band gap of CYO:Ho^3+^,xEu^3+^ (where x = 0, 1, 3, 5, 7, 9 mol%) nanophosphor samples have been estimated using Tauc’s relation (3) given in Fig. 5b^14^:

3 $$\alpha h\nu =A{(h\nu -{E}_{g})}^{n}$$

Here, *hν* is the energy of the incident photon, α is the absorption coefficient, *E*_*g*_ is the energy of the optical bandgap, and A represents a constant known as band tailoring parameter. The value of ‘n’ is different for different transitions as ½ for direct allowed, 3/2 for direct forbidden, 2 for indirect allowed and 3 for indirect forbidden transition, respectively. Here, we consider direct allowed transition so in the present case value of ‘n’ has been chosen as 1/2. The value of the optical bandgap for direct allowed transition has been calculated by extrapolating the linear region of the plot up to α = 0. The calculated value of optical band gap (E_g_) for CYO:Ho^3+^,xEu^3+^ with x = 0, 1, 3, 5, 7, 9 mol% nanophosphor samples have been found as 5.547, 5.539, 5.481, 5.549, 5.556, 5.565 eV, respectively. It has been realized that the optical band gap decreases with doping up to 5 mol% and further increases, this variation may be associated with the change in the local environment of lattice due to doping^41^.

### Luminescence properties of CYO:Ho^3+^,xEu^3+^

**Figure 6:**
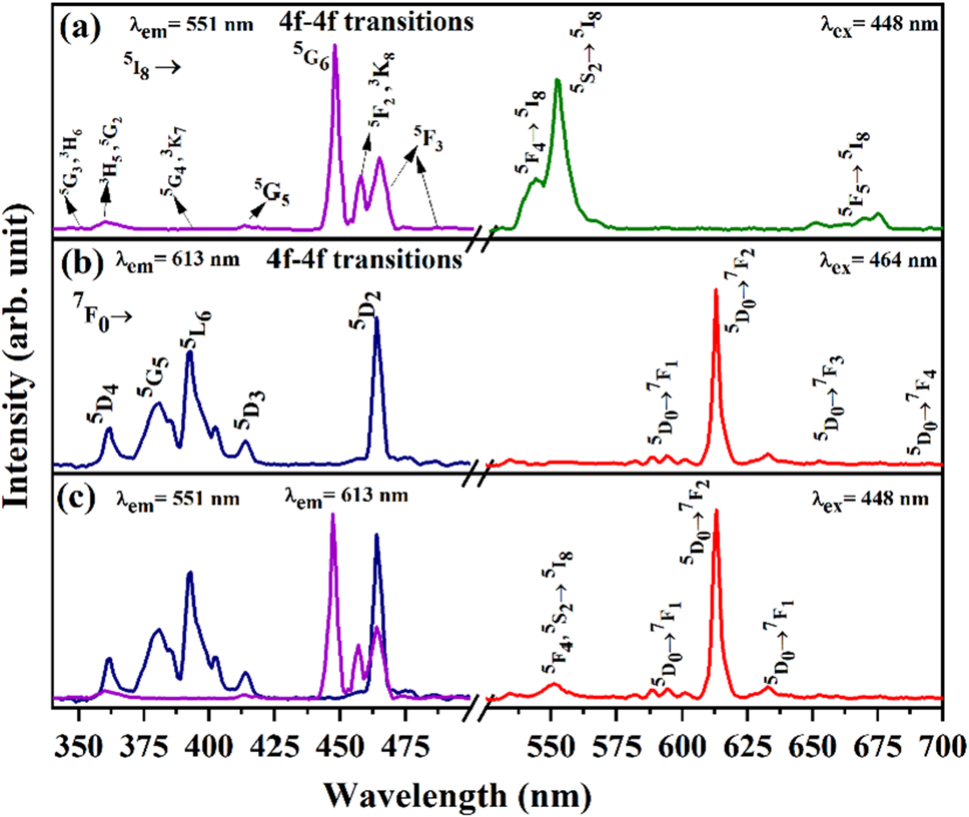
Excitation and emission spectra of (**a**) CYO:Ho^3+^ (2.0 mol%) (**b**) CYO:Eu^3+^ (2.0 mol%) and (**c**) CYO: (2.0 mol%) Ho^3+^,xEu^3+^ (Eu^3+^ 5.0 mol%) nanophosphor.

Figure 6a describes the photoluminescence excitation and emission spectra of CYO:Ho^3+^(2.0 mol%) nanophosphors. The excitation spectra have been recorded in the range 340–500 nm, which exhibits several narrow peaks with an intense peak at ~ 448 nm. The appearance of these peaks is due to the 4f.-4f. transition of Ho^3+^ ions. The excitation takes place from the ground state (← ^5^I_8_) to several excited states at 346 nm (^5^G_3_,^3^H_6_ ← ^5^I_8_), 360 nm (^3^H_5_,^5^G_2_ ← ^5^I_8_), 387 nm (^5^G_4_,^3^K_7_ ← ^5^I_8_), 417 nm (^5^G_5_ ← ^5^I_8_), 448 nm (^5^G_6_ ← ^5^I_8_), 455 nm and 463 nm (^5^F_2_,^3^K_8_ ← ^5^I_8_), 487 nm (^5^F_3_ ← ^5^I_8_) transitions, respectively^12^. Excitation spectra possess similar to the absorption spectra (Fig. 5a). Among all the transitions, 448 nm (^5^G_6_ ← ^5^I_8_) is the most intense peak, hence it has been taken as excitation wavelength for recording PL spectra. The PL emission of CYO:Ho^3+^(2.0 mol%) excited by blue light (λ_ex_ = 448 nm) was recorded in the region 525–700 nm. CYO:Ho^3+^ doped nanophosphor spectra display the presence of three emission transitions. There is an intense green emission peak at 551 nm (^5^F_4_, ^5^S_2_ → ^5^I_8_) with low-intensity peaks 541 nm (^5^F_4_, → ^5^I_8_), and 654 nm (^5^F_5_ → ^5^I_8_) correspond to green and red emission in visible light spectra, respectively. These less intense peaks correspond to the splitting of the stark level of ^5^F_4_, ^5^S_2_ levels of Ho^3+^ ions in the crystal field of CYO^42^.

Figure 6b show the PL excitation and emission spectra of CYO: xEu^3+^ (5.0 mol%) recorded for the 613 nm wavelength in the 340–500 nm range. The spectra consist of several excitation peaks corresponds to 361 nm (^7^F_0_ → ^5^D_4_), 379 nm (^7^F_0_ → ^5^L_7_), 393 nm (^7^F_0_ → ^5^L_6_), 413 nm (^7^F_1_ → ^5^D_3_) and 464 nm (^7^F_0_ → ^5^D_2_) respectively, which correspond to the 4f.-4f. transition of Eu^3+^ ions in CYO host. It has been noted that the nanophosphor is effectively excited by the near-UV region. As spectra show maximum excitation at 464 nm (^7^F_0_ → ^5^D_2_), it has been taken for monitoring the emission spectra^30,43^. The emission spectra of CYO:Eu^3+^ is composed of four characteristic emissions of Eu^3+^ ions corresponding to 593 nm (^5^D_0_ → ^7^F_1_), 612 nm (^5^D_0_ → ^7^F_2_), 653 nm (^5^D_0_ → ^7^F_3_), 698 nm (^5^D_0_ → ^7^F_4_) transitions respectively. It is well known that the symmetry position of the Eu^3+^ ion in the host can influence the characteristic emission. If the Eu^3+^ ions are located at the inversion symmetric site in the Ca-doped Y_2_O_3_ host (CYO), the magnetic dipole transition (^5^D_0_ → ^7^F_1_) dominates. When Eu^3+^ ions are located at the antisymmetric site (^5^D_0_ → ^7^F_2_), it promotes magnetic dipole transition. It can be seen from Fig. 6b that the strongest peak located at 613 nm (^5^D_0_ → ^7^F_2_) peaks correspond to red emission. So, Eu^3+^ ions are located at anti-symmetry sites of the host lattice^44^.

Figure 6c depicts the PL excitation and emission spectra of CYO:Ho^3+^,xEu^3+^ nanophosphor. The PL excitation spectra have been monitored with 551 nm and 612 nm have been found identical to those of singly doped Ho^3+^ and Eu^3+^. So, the doubly doped CYO:Ho^3+^,xEu^3+^ may be used for double-colour-emitting nanophosphor which emit green and red colour used for white LEDs. It can further be seen that both the green and red emissions can be observed under 464 nm excitation of Ho^3+^, which signifies the energy transfer from Ho^3+^  → Eu^3+^. Moreover, as the nanophosphors emit both green emission of Ho^3+^ ion and red emission of Eu^3+^ ion, so the colour of nanophosphors can be tuned by monitoring the doping strategy ratio of Ho^3+^ and Eu^3+^ concentrations.

**Figure 7:**
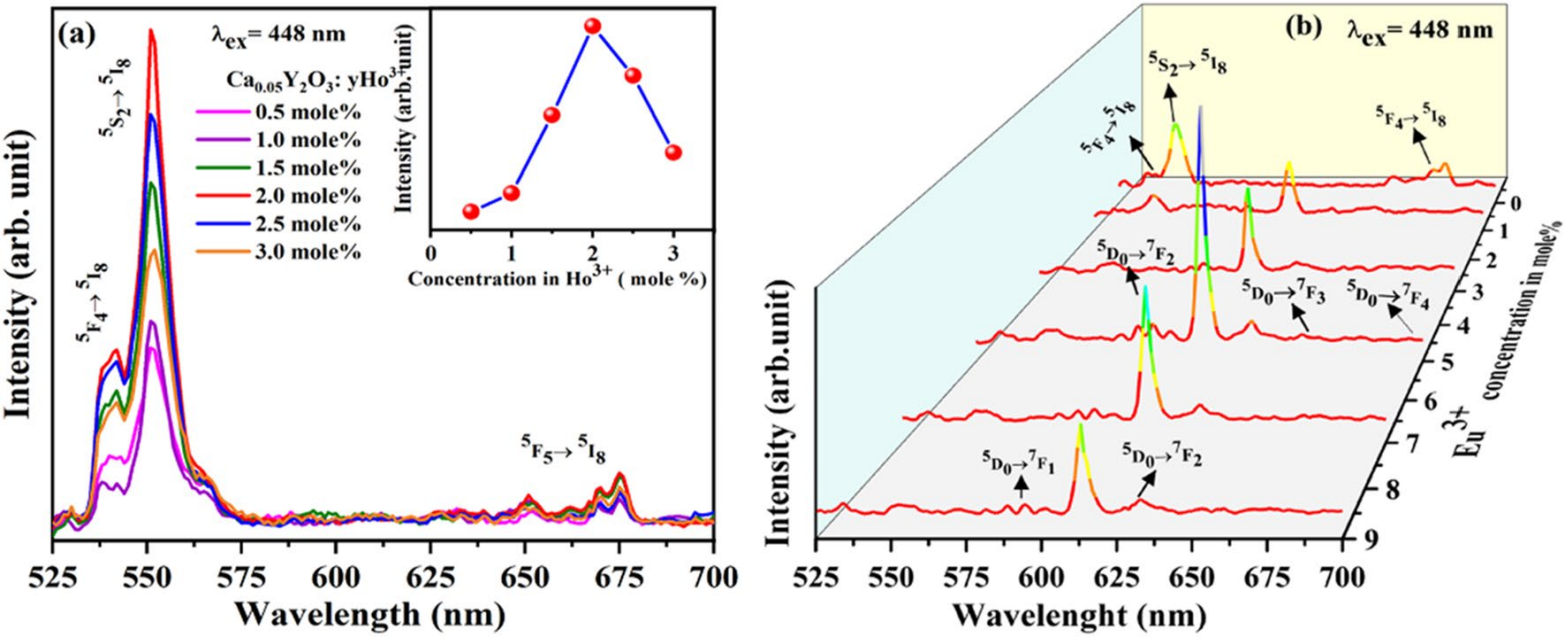
(**a**) Photoluminescence emission spectra of CYO:Ho^3+^ (0.05, 1.0, 1.5, 2.0, 2.5, 3 mol%) (inset: effect on PL emission intensity with concentration of Ho^3+^ ions) (**b**) Emission spectra of CYO:2.0 Ho^3+^,xEu^3+^ (x = 0, 1, 3, 5, 7, 9 mol%) at λ_ex_ = 448 nm.

With this the effect on PL emission intensity with doping concentration of Ho^3+^ ions (0.05, 1.0, 1.5, 2.0, 2.5, 3 mol%) in CYO host has also been also observed and presented in Fig. 7a. It can be seen form inset of Fig. 7a that the PL intensity increases up to 2.0 mol% doping and further has a decreasing trend. This is due to effect of concentration quenching^14^. Hence, the 2 mol% doped CYO:Ho^3+^ has been consider for further doping with Eu^3+^, in the formation of Y_2_O_3_:0.02Ho^3+^,0.05Eu^3+^nanophopher. Moreover, to understand the effect of Eu^3+^ ion concentrations on the emission intensity of CYO:Ho^3+^,xEu^3+^ nanophosphor, we symmetrically recorded PL spectra of CYO:Ho^3+^,xEu^3+^ (x = 0, 1, 3, 5, 7, 9 mol%) with 448 nm excitation as shown in Fig. 7(b). It can be seen that with the increasing concentration of Eu^3+^ ions, the emission intensity of Eu^3+^ ions increase, while that of Ho^3+^ ion decreases gradually. Thus, tuning of the colour could be realized by changing the doping concentration ratio of Ho^3+^ and Eu^3+^.

**Figure 8:**
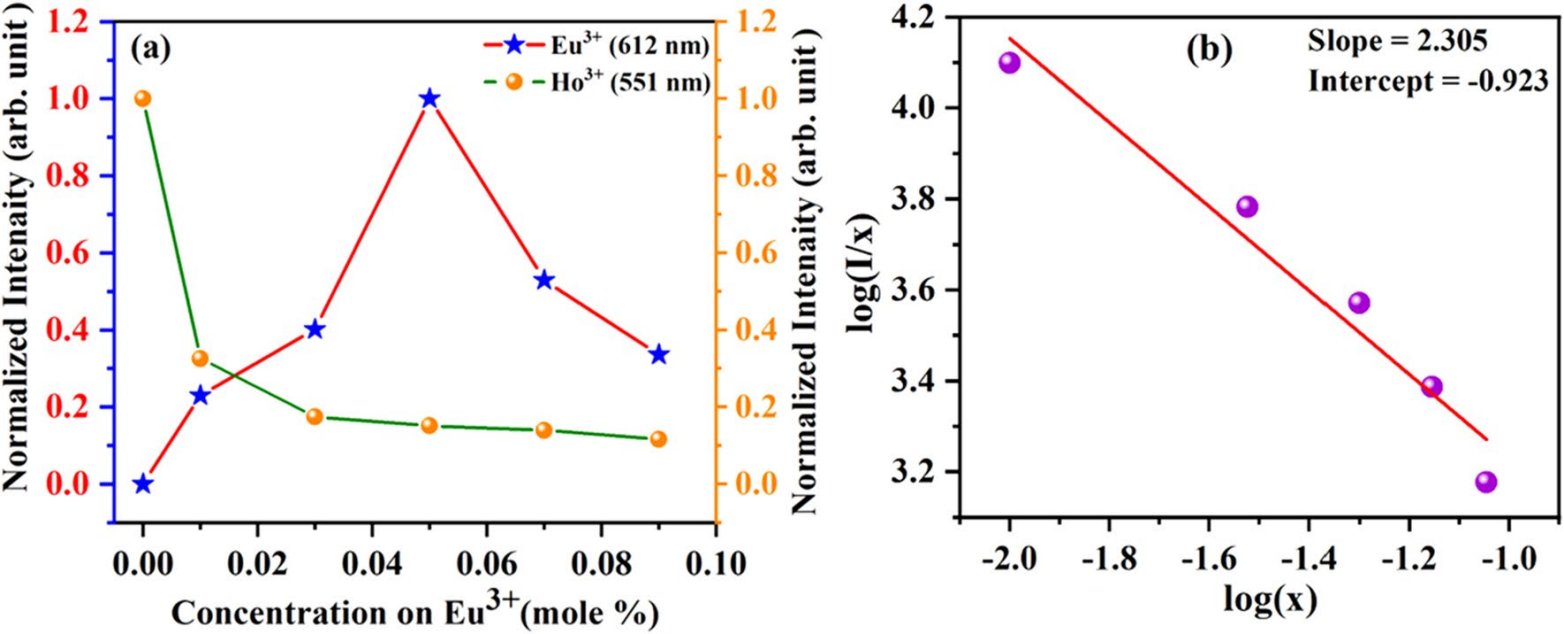
(**a**) variation of Eu^3+^ ion concentration with the Eu^3+^ emission and Ho^3+^ emission in CYO:Ho^3+^,xEu^3+^ (x = 0, 1, 3, 5, 7, 9 mol%) nanophosphor (**b**) The variation of log(x) versus log(I/x) of CYO:Ho^3+^,xEu^3+^ nanophosphor.

To understand the relative emission intensity variation, the intensity of Ho^3+^ and Eu^3+^ emission with Eu^3+^ doping concentration (x = 0, 1, 3, 5, 7, 9 mol%) in CYO:Ho^3+^,xEu^3+^ nanophosphor has been presented in Fig. 8a. It shows that with increasing concentration of Eu^3+^ ions from 1–9 mol%, the green emission of Ho^3+^ ions decrease gradually due to energy transfer from Ho^3+^  → Eu^3+^, whereas the characteristic red emission of Eu^3+^ increases gradually from 1 to 5 mol% and further decreases due to the concentration quenching effect. A similar observation has also been reported by Devakumar et al.^45^. Hence, the concentration quenching occurs due to energy transfer and can be explained with the help of Dexter theory given in Eq. (4)^44,45^.

4 $$\frac{I}{X} = K\left[ {1 + \beta \left( X \right)^{\frac{Q}{3}} } \right]^{ - 1}$$

where I refers to the PL emission intensity, X is doping concertation of the activator (Eu^3+^) ion, k & β are constants for a given host and Q stands for the multipole interaction. Q represents the multipole nitration and can have values as Q = 6, 8 and 10 for dipole–dipole, dipole-quarter pole and quadrupole–quadrupole interactions, respectively^44^. The plot between log(I/x) and log(x) for CYO:Ho^3+^,xEu^3+^ (x = 0, 1, 3, 5, 7, 9 mol%) has been given in Fig. 8b It can be perceived that log(I/x) varies linearly with the log(x) and slope value Q has been found as ⁓ 2.76, which is close to 6 and stipulates energy transfer occurs due to dipole–dipole interaction.

### Decay curves and energy transfer of Y_2_O_3_:Ho^3+^, Eu^3+^

**Figure 9:**
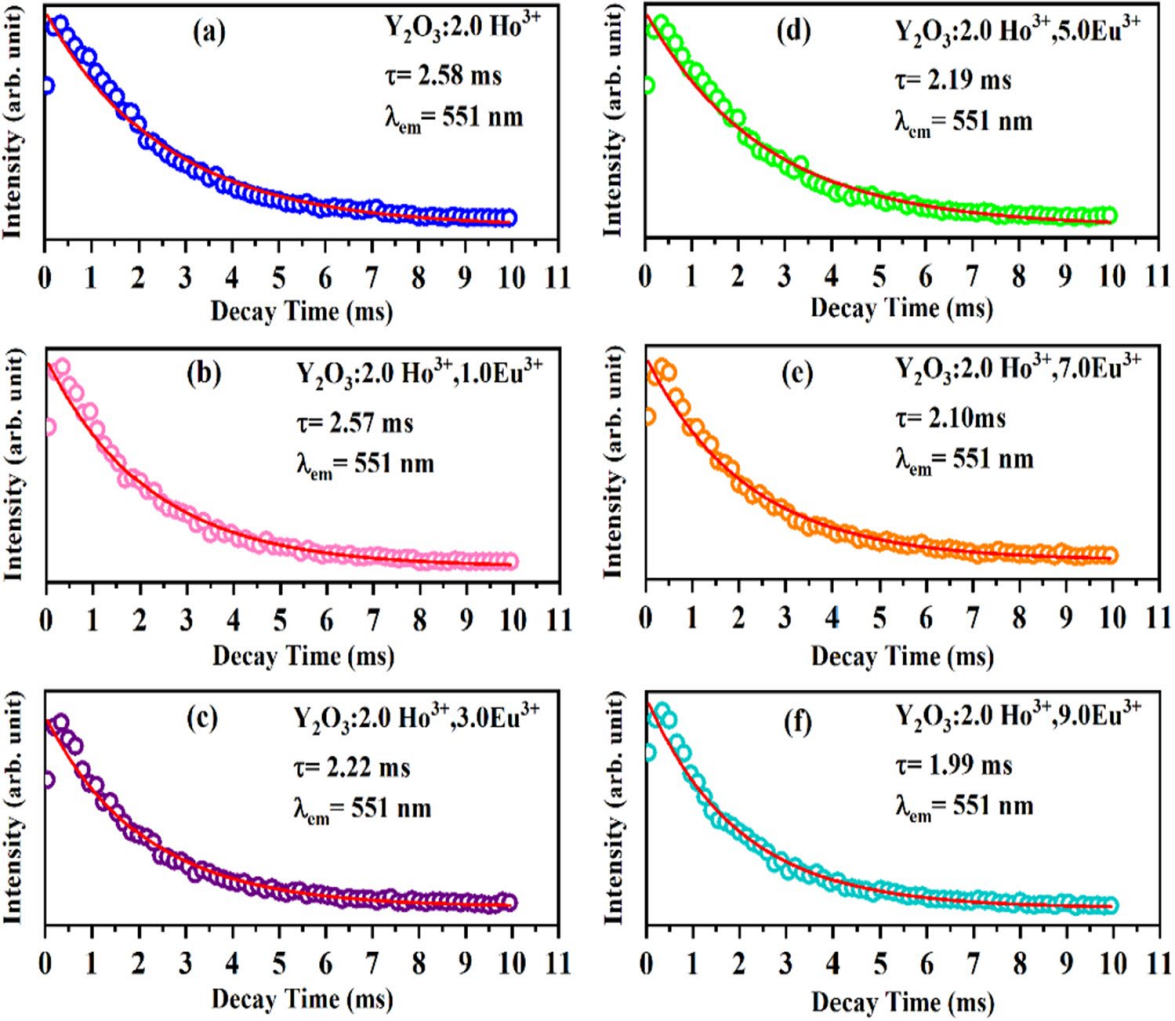
(**a-f**) Lifetime decay curves of Y_2_O_3_:2.0 Ho^3+^, xEu^3+^ (0, 1, 3, 5, 7, 9 mol%) excited by 448 nm and monitored at 551 nm (^5^S_2_ → ^5^I_8_).

To identify the energy transfer from Ho^3+^  → Eu^3+^ ions, the decay curves of CYO:Ho^3+^,xEu^3+^ nanophosphor have been dignified with an excitation wavelength ~ 448 nm as shown in Fig. 9a-f. The decay curve has been found to fit well with a single-exponential equation and the corresponding lifetime value has been calculated by employing the following Eq.^46^:

$$I(t) = I_{0} e^{{ - \frac{t}{\tau }}} ,$$

where I (t) represent intensity at time and I_0_ at time t = 0 s and t are the lifetime of ^5^S_2_ level of Ho^3+^ ions. The life time value of 551 nm (^5^S_2_ → ^5^I_8_) excited level of CYO:Ho^3+^,xEu^3+^ (0, 1, 3, 5, 7, 9 mol%) nanophosphor have been evaluated as 2.58, 2.57, 2.22, 2.19, 2.10, 1.99 ms, respectively. It shows that the lifetime values decrease with increasing doping concentration of Eu^3+^ ions, which confirm the interionic energy transfer. Energy transfer might also be due to the existence of additional decay levels created through doping.

### Energy transfer

**Figure 10:**
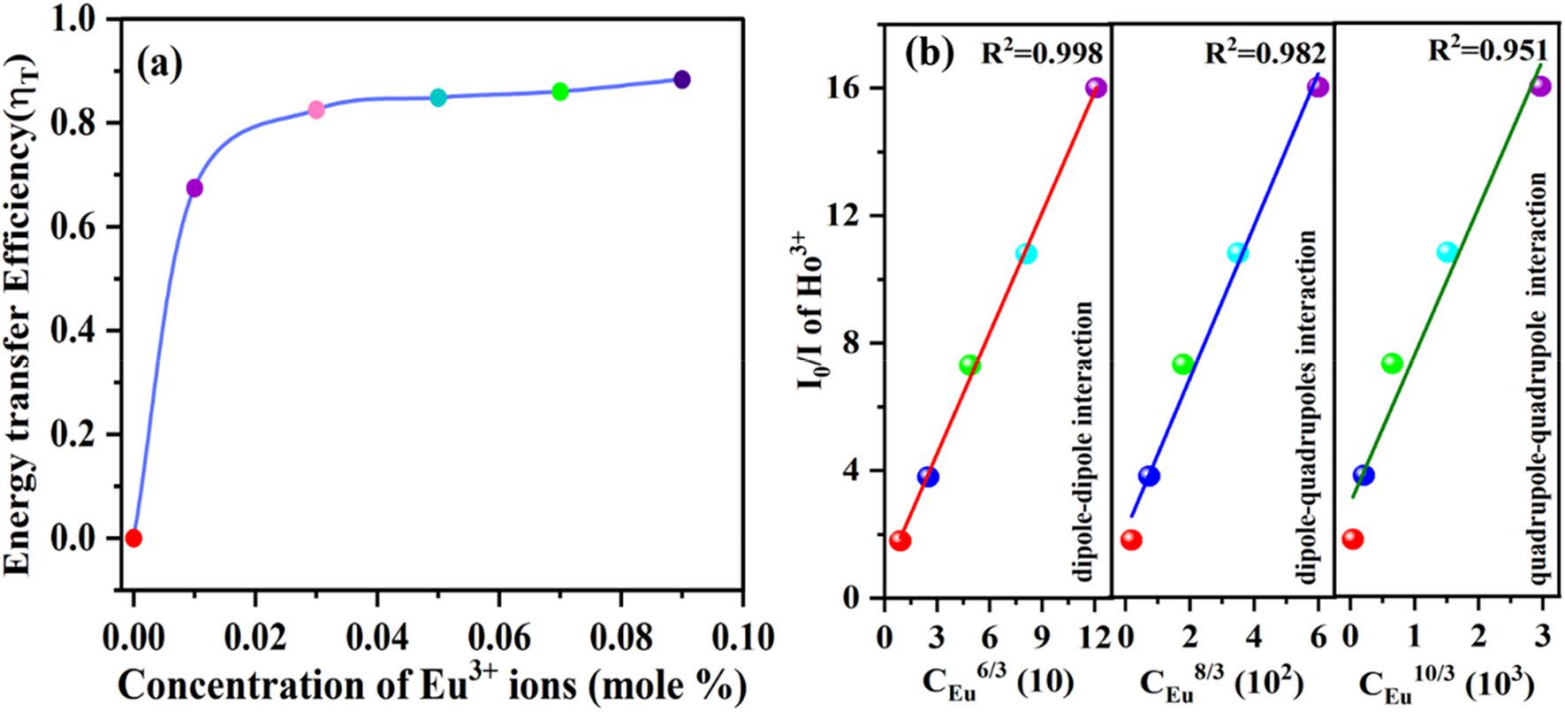
(**a**) The energy transfer efficiency of from Ho^3+^ → Eu^3+^ in CYO:Ho^3+^,xEu^3+^ (x = 0, 1, 3, 5, 7, 9 mol%) nanophosphor at 448 nm excitation wavelength (**b**) Dependence of *I*_*0*_*/I* on *C*^*6/3*^*, C*^*8/3*^ and *C*^*10/3*^ for CYO:Ho^3+^,xEu^3+^ (x = 0, 1, 3, 5, 7, 9 mol%) nanophosphors.

Furthermore, the energy transfer efficiency (η_T_) from Ho^3+^ → Eu^3+^ in CYO:Ho^3+^,xEu^3+^ nanophosphor can be estimated using the Eq. (5) ^2,47^:

5 $${\eta }_{T}=1-\frac{I}{{I}_{0}}$$

where η_T_ represents the energy transfer efficiency, *I*_0_ & *I* correspond to the PL emission intensities of the sensitizer (Ho^3+^ ions) without and with acceptor ion (Eu^3+^ ions), respectively given in Fig. 10a. It can be seen that with increasing the doping concentration of Eu^3+^, the η_T_ value gradually increases and reaches up to 88.6% with 9 mol% doping.

In general, the resonance energy transfer mechanism from sensitizers (Ho^3+^) to activator (Eu^3+^) in nanophosphors can be through the major and foremost processes, the exchange interaction processes and multipolar interaction^45,46^. The exchange interaction primarily depends on the critical distance between the donor (Ho^3+^) and the acceptor (Eu^3+^). The exchange interaction is prominent if the critical distance between the respective ions (R_c_) is less than 5 Å whereas, R_c_ greater than 5 Å leads to the multipole interactions. The distance between Ho^3+^ to Eu^3+^ ions can be calculated according to Blasses’s theory through the Eq. (6)^45,48^:

6 $$R_{c} \approx 2\left[ {\frac{3V}{{4\pi X_{c} Z}}} \right]^{1/3}$$

where R_c_ is the critical distance, V represents the unit cell volume, ${X}_{c}$ is optimum concertation of dopant ion for which the PL intensity of the activator is half of the sample without dopant and Z is the number of sites that dopant can occupancy per unit cell in the host lattice. For the CYO host, Z = 24, V = 1194.69, *X*_*c*_ = 0.0274. Therefore, the estimated critical distance for energy transfer comes out to be 15.146 Å, which is larger than 5 Å. This is indicative of the multipole—multipole interaction contribution for the energy transfer from Ho^3+^ to Eu^3+^ions. The multipole interaction can be determined according to Dexter formula for energy transfer and Reisfield’s approximation: $\frac{{I}_{0}}{I} \propto {C}^\frac{n}{3}$ where I and I_0_ represent the emission intensities of the donor (Ho^3+^) in the presence and absence of acceptor (Eu^3+^), C is the sum of the doping concentration of Ho^3+^ and Eu^3+^ and n = 6, 8 and 10 corresponding to dipole–dipole, dipole-quadrupoles and quadrupole–quadrupole interaction, respectively^45,46^. The plot of I_0_/I of has been presented in Fig. 10b, the better linear behaviour has been achieved for n = 6, signifying that energy transfer from Ho^3+^ to Eu^3+^ takes place via electric dipole–dipole interaction.

**Figure 11:**
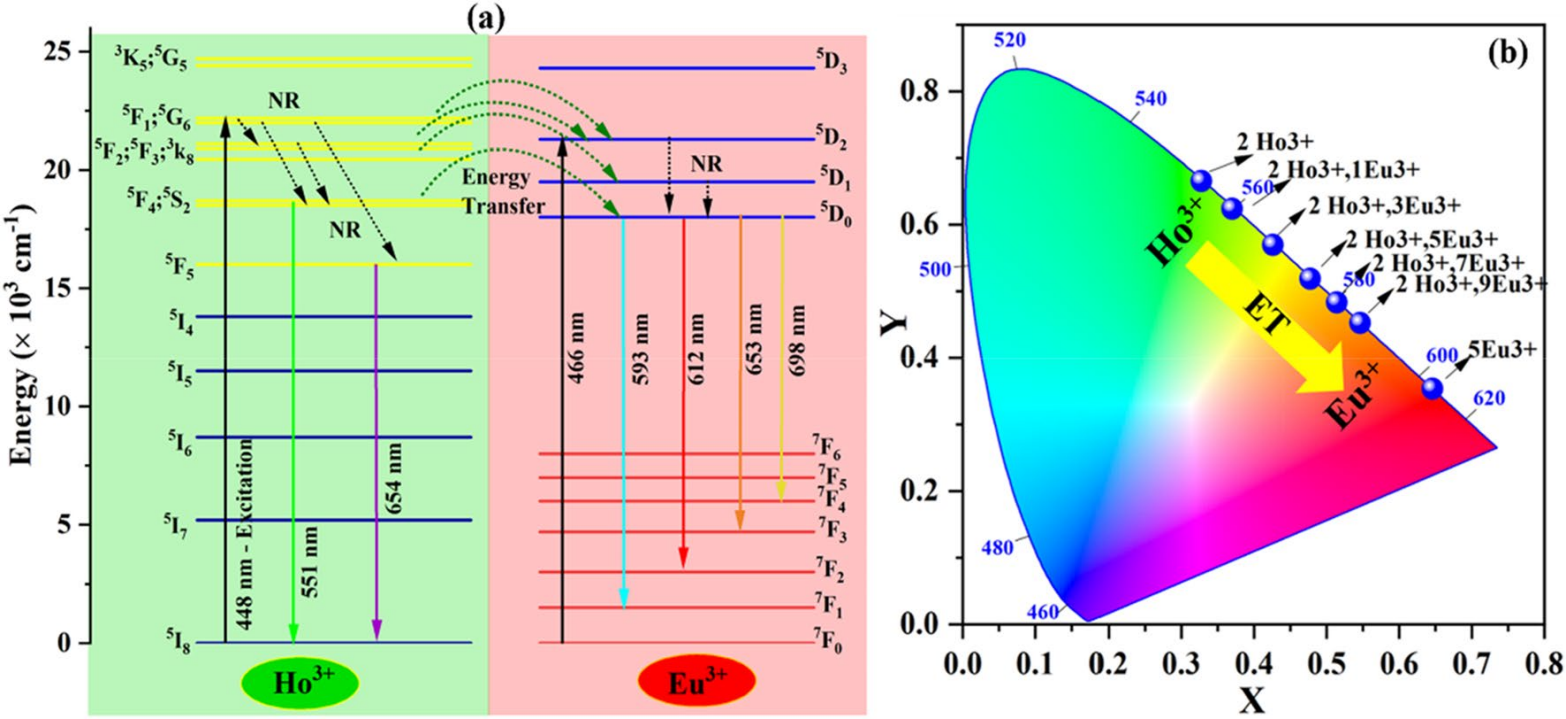
(**a**) Partial energy level diagram of Ho^3+^ and Eu^3+^ and energy transfer schematic of CYO:Ho^3+^,Eu^3+^ nanophosphor (**b**) The Commission International de L’Eclairage (CIE) chromaticity coordinates of CYO:Ho^3+^,xEu^3+^ (0, 1, 3, 5, 7, 9 mol%) nanophosphor sample.

Figure 11a illustrate the partial energy level diagram of Ho^3+^ and Eu^3+^ in CYO:Ho^3+^,xEu^3+^ nanophosphor. Under the excitation of 448 nm wavelength, the electron absorbs energy and get excited to the higher excited states of Ho^3+^ and Eu^3+^ of ^5^G_6_, ^5^F_2_, ^3^K_8_ and ^5^D_2_, respectively. These excited electrons relax to the next energy level through non-radiative transitions. Finally, the excited electrons fall to the ground state by emitting green, and red emissions through 551 nm (^5^F_4_, ^5^S_2_ → ^5^I_8_), 541 nm (^5^F_4_, → ^5^I_8_), and 654 nm (^5^F_5_ → ^5^I_8_) transition of Ho^3+^ and 593 nm (^5^D_0_ → ^7^F_1_), 612 nm (^5^D_0_ → ^7^F_2_), 653 nm (^5^D_0_ → ^7^F_3_), 698 nm (^5^D_0_ → ^7^F_4_) transitions of Eu^3+^ ions. When Eu^3+^ is doped with the CYO:Ho^3+^, a part of energy transfer occurs from ^5^G_6,_^5^F_4_, ^5^S_2_ states of Ho^3+^ to ^5^D_1_, ^5^D_0_ states of Eu^3+^ resulting in the decrease of green emission of Ho^3+^ and increase of red emission of Eu^3+^. However, with the increasing concentration of Eu^3+^, the cross-relaxation process occurs and leads to a decrease in the emission intensity.

### CIE chromaticity coordinates and colour purity

The CIE (Commission International de L’Eclairage) coordinate of the CYO:Ho^3+^,xEu^3+^ nanophosphor have been estimated from 1931CIE. The CIE diagram for the excitation wavelength 448 nm has been represented in Fig. 11b and the respective estimated values have been put forward in Table 2. For the 0 mol% doping in CYO:Ho^3+^,xEu^3+^ the emission color of nanophosphor exhibits green in color (0.327 0.665). It can also be seen that, with increasing the doping concentration of Eu^3+^, the CIE coordinate of CYO:Ho^3+^,xEu^3+^ (0, 1, 3, 5, 7, 9 mol%) nanophosphor gets changed from green (0.327 0.665) for x = 0 mol% to red color (0.545 0.452) x = 9 mol%. The remarkable change in emission colour presents the possibility of multicolour tunability with a varying doping concentration of Eu^3+^ions. Hence the CYO:Ho^3+^,xEu^3+^ may be a pertinent candidate with potential prospects for multicolour- display and lighting applications^46,49^.

**Table 2:** The CIE coordinates, CCT coordinate, CCT values, % color purity and CIR value of CYO:Ho^3+^,xEu^3+^ (0, 1, 3, 5, 7, 9 mol%) nanophosphors.

CYO:Ho^3+^,xEu^3+^ Nanophosphor (at. mole%)	CIE coordinates (*x*, *y*)		CCT (U’, V’)		CCT value (K)	Color purity (%)	CRI
0	0.327	0.665	0.126	0.579	5586	99	98.4
1	0.369	0.625	0.157	0.576	4960	95	98.6
3	0.425	0.569	0.189	0.570	4058	83	98.8
5	0.477	0.518	0.230	0.564	3178	77	98.9
7	0.513	0.483	0.264	0.559	2564	77	98.9
9	0.545	0.452	0.297	0.555	2084	79	98.8

Customarily, the quality of a light source can be accessed in the form of correlated colour temperature (CCT), and the respective values can be evaluated through McCamy’s empirical formula as given below (Eq.9)^46,50^ and respective estimated values are summarized in Table 2.

7 $${U}^{^{\prime}}=\frac{4x}{-2x+12y+3}$$

8 $${V}^{^{\prime}}=\frac{9x}{-2x+12y+3}$$

9 $$T=-499{n}^{3}+3525{n}^{2}-6823.3n+5520.33$$

where $n=\frac{x-0.332}{y-0.186}$ and (x, y) are CIE coordinates. The estimated values of CYO:Ho^3+^, xEu^3+^ (0, 1, 3, 5, 7, 9 mol%) nanophosphor samples as listed in Table 2 suggests it to lie in the range 2084–4960 K, subjected to the different Eu^3+^ doping concentrations. It has been reported that if the CCT has a value less than 5000 K, it would be appropriate for the warm red light pertinent to the solid-state lighting applications^46^. With this, the CIE value for CYO:Ho^3+^ nanophosphor lie in the green region and CCT has been found as 5224 K. Additionally, the colour purity is also one of the important parameters for the WLED application. The colour purity has been calculated using the following relation (Eq.10) for the nanophosphor samples^45,46^.

10 $$Color \, purity = \frac{\sqrt{\surd {(x-{x}_{i})}^{2}+{(y-{y}_{i})}^{2}}}{\sqrt{\surd {({x}_{i}-{x}_{d})}^{2}+{({y}_{i}-{y}_{d})}^{2}}} \times 100 \%$$

where (*x,y*), (${x}_{i}, {y}_{i}$) and (${x}_{d}$, ${y}_{d}$) represent the CIE coordinates of the sample point, standard source, and dominant wavelength of the sample, respectively. The value of (${x}_{i}, {y}_{i}$) was (0.33, 0.33) and the calculated values of colour purity for samples have also been presented in Table 2. It can be observed that the colour purity of CYO:Ho^3+^,xEu^3+^ sample recline in the region 98.6–98.9. High colour purity value significantly suggests that the as-synthesised samples have great promise in WLEDs- application Hence the CYO:Ho^3+^,xEu^3+^ may be a pertinent candidate with potential prospects for multicolour- display and lighting applications^46,49^.

### Thermal stability and quantum efficiency (QE)

**Figure 12:**
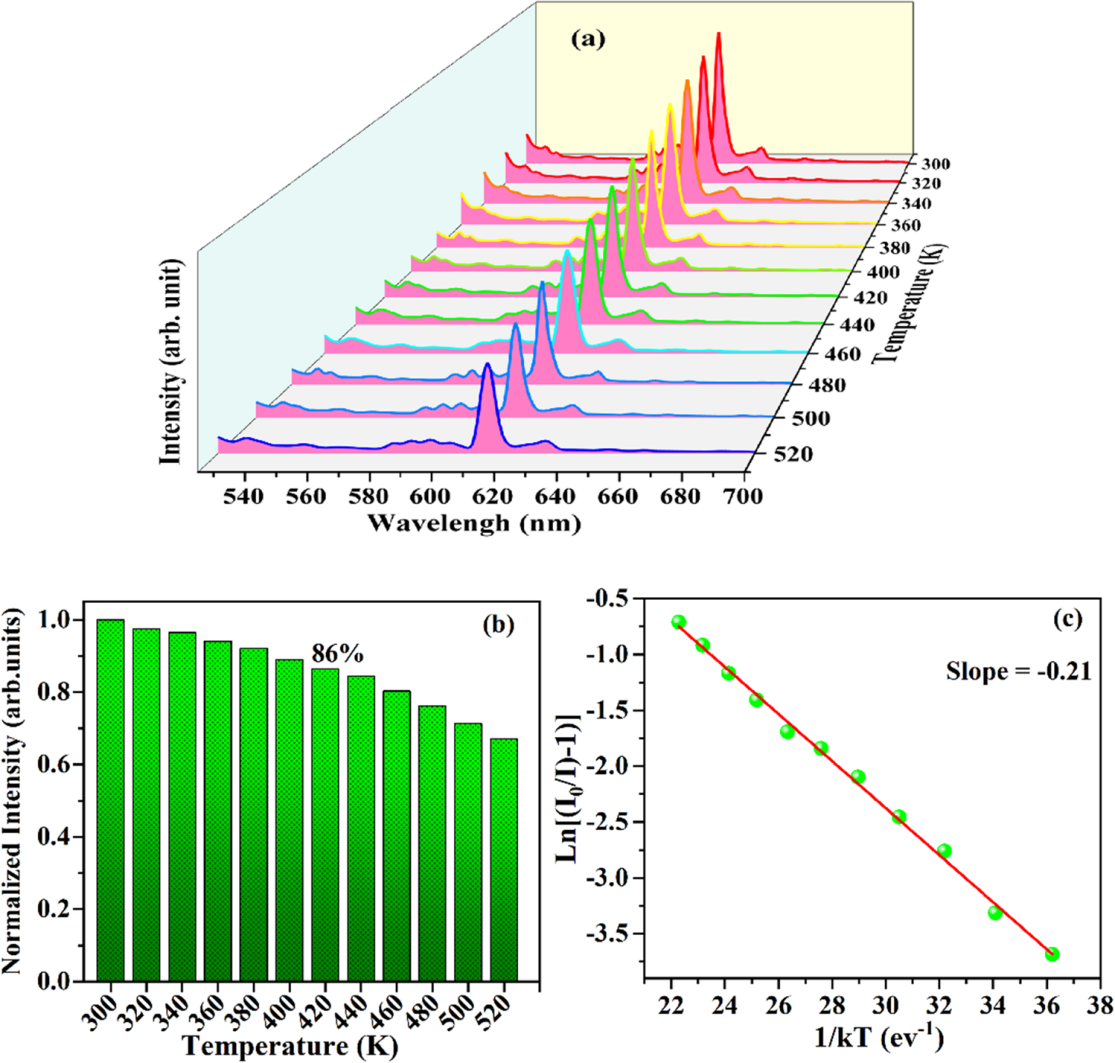
(**a**) PL emission spectra as a function of temperature (**b**) normalized PL emission intensity with different temperature (**c**) Arrhenius plot of ln [(I_0_/I)-1] versus 1/kT for thermal quenching nature, of CYO: Ho^3+^,0.05 Eu^3+^ sample.

Thermal stability has been one of the significant parameters of nanophosphor materials for LEDs for industrial applications as it offers a high impact on the CIE, lifetime, and output lights of the LEDs. Figure 12 displays the temperature dependent emission spectra of CYO: Ho^3+^,0.05 Eu^3+^ nanophosphor measured under the excitation 448 nm in the 300–520 K range. It reveals that there has been no effect on the profile of the PL spectra with the increasing temperature, but intensity of the PL emission gradually decreases. This might be due to thermal quenching owing to a non-radiative phonon relaxation form higher levels via crossover process. Furthermore, the PL intensity of the CYO: Ho^3+^,0.05Eu^3+^ nanophosphor at 420 K remains 86% of the initial temperature 300 K. The other Eu^3+^ doped nanophosphors such CAZO:Bi^3+^, Eu^3+^, LiGd(WO_4_)_2_: Eu^3+^, and Ca_3_Gd(AlO)_3_(BO_3_)_4_:Tb^3+^, Eu^3+^ exhibit thermal stability as 80%, 81% and 83%, respectively^9,10^. It shows that CYO: Ho^3+^, 0.05Eu^3+^ nanophosphor possesses better thermal stability. Further, the activation energy for the thermal quenching can be estimated through Arrhenius Eq. (11)^3,51^:

11 $$\frac{I}{{I}_{0}}={[1+Aexp\left(\frac{{E}_{a}}{kT}\right)]}^{-1}$$

where I_0_ is initial emission intensity, and I represent intensity at temperatures T, k is Boltzmann constant, A is constant and E_a_ is activation energy respectively. As reported by many researchers, activation energy E_a_ is the energy required to raise the electron from relaxed excited level to the host lattice conduction band^10,52^. Heating at higher temperature provides this amount of energy and increases the probability of manifestation of this process to decrease of PL emission intensity nanophosphor. From the Arrhenius equation, ln [(I_0_/I)-1] versus 1/kT plot has been drawn and fitted lineally with the slope −0.21 eV. The slope gives activation energy, therefore E_a_ the for the thermal quenching has been estimated as 0.21 eV, which is higher than the commercial Y_2_O_3_:Eu^3+^ phosphor (⁓0.17 eV)^53^. Hence, the CYO: Ho^3+^, 0.05 Eu^3+^ nanophosphor has good thermal stability and can be used for the for LEDs fabrication.

To examine the luminescence performance for commercial application of CYO: Ho^3+^, 0.05 Eu^3+^ sample we have estimated the quantum efficiency (QE) of as-synthesized nanophosphor using following formula (12) ^10,54^:

12 $${\eta }_{QE}=\frac{\int {L}_{s}}{\int {E}_{R }-\int {E}_{S}}$$

where η_QE_ represents the quantum efficiency L_S_ is the integrated emission spectrum of the sample, E_R_ and E_S_ denote the integrating sphere of excitation light with and without the sample, respectively. The QE of the sample with λ_ex_ = 448 nm excitation wavelength has been evaluated by integrated emission count in the 500–700 nm range. The value of QE for CYO: Ho^3+^,xEu^3+^ (1, 3, 5, 7, 9 mol%) has found as 66.4%,70.1%, 83.6%, 74.9%, 79.2% and 68.4%, respectively. The obtained QE value in the present study has been much higher than the value reported for Ca-doped Eu:Y_2_O_3_ (⁓69%)^21^, Ca-Eu:Y_2_O_3_ (81%)^29^ and Ca_0.5_Y_1.90-x_O_3_:Ho^3+^ (81.1%)^55^. It is noticeable that the QE of CYO: Ho^3+^, xEu^3+^ is higher than the bare Eu^3+^ / Ho^3+^ doped samples. This might be due to the energy transfer from Ho^3+^ → Eu^3+^ by enhancing the concentration of luminescence centre through co-doping^9^. The higher value (83.6) of QE specifies that the CYO: Ho^3+^,0.05Eu^3+^ nanophosphor would be a promising for the LEDs application.

## Conclusion

A series of green to red colour tunable CYO:Ho^3+^,xEu^3+^ nanophosphors have been successfully synthesised through a facile solution combustion method. The XRD pattern and Rietveld refinement confirm that the as-prepared nanophosphor exhibits a crystalline nature with cubic phase. TEM analysis indicates the irregular morphology with agglomeration in the nanometre range. Under 448 nm excitation, nanophosphors show intense green emission to red emission of Ho^3+^/Eu^3+^ ions due to 4f.-4f. transition. It has been seen that regulating the doping ratio of Ho^3+^/Eu^3+^, as-synthesized nanophosphor manifests multicolour tunability from green-yellow to red. Emission spectra and decay lime time curve suggest dipole–dipole interaction causes energy transfer from Ho^3+^ → Eu^3+^. CIE coordinate illustrates that the singly doped Ho^3+^ and Eu^3+^ lie in the green and red region, respectively, while the as-synthesized CYO:Ho^3+^,xEu^3+^ shows tunability from green to red. Furthermore, the temperature dependent emission spectra of CYO:Ho^3+^,xEu^3+^ nanophosphor demonstrates a high thermal stability with an activation energy ⁓ 0.21 eV, and the quantum efficiency of 83.6%. Henceforth, the as-synthesized CYO:Ho^3+^,xEu^3+^ nanophosphor can be used as a near-UV excited multicolour colour-tunable entrant for multicolour- display, optoelectronic devices and lighting applications.

## Experimental section

### Materials

Yttrium oxide (Y_2_O_3_), Europium oxide (Eu_2_O_3_), Holmium oxide (Ho_2_O_3_) calcium oxide (CaO) and nitric acid (HNO_3_) were purchased from Sigma-Aldrich. Urea (NH_2_CONH_2_) was purchased from Molychem. All the chemical reagents were of analytical grade and used in the synthesis without any further purification.

### Materials Synthesis

A low-temperature facile solution combustion method was used for the synthesis of a series of Eu^3+^-doped Ca_0.05_Y_1.93-_xO_3_:0.02Ho^3+^ (CYO:Ho^3+^,xEu^3+^) nanophosphors. This technique has been advantageous over other typical procedures due to some peculiarities, such as an even mixing, low processing temperature, and small energy consumption during the synthesis of nanophosphor samples. Briefly, the starting materials Eu_2_O_3_, Ho_2_O_3_, Y_2_O_3_ and CaO were dissolved in 2 ml concentrated nitric acid with 10 ml deionised water in a beaker. The composition of the compounds has been taken as:

$$\left( {100 - {\text{x}} - {\text{y}} - {\text{z}}} \right){\text{Y}}_{2} {\text{O}}_{3} + {\text{xEu}}_{2} {\text{O}}_{3} + {\text{yHo}}_{2} {\text{O}}_{3} + {\text{zCaO}} + {\text{NH}}_{2} {\text{CONH}}_{2} \to {\text{Eu}}_{{\text{x}}} {\text{Ho}}_{{\text{y}}} {\text{Ca}}_{{\text{z}}} \cdot {\text{Y}}_{2 - (x + y + z)} {\text{O}}_{2} + {\text{water}}\;{\text{vapour}} + {\text{gases }}\left( {\mathbf{1}} \right)$$

where x = 0, 1, 3, 5, 7, 9 mol%, y = 2.0 mol%, z = 5 mol%

Further, the as-received solution containing nitrate of the compounds was mixed with the 3 gms of urea which act as a reducing agent dissolved in 5 ml of deionised water, followed by a continuous stirring of 1 h at 40 °C to get a transparent solution. Next, the obtained solution was transferred to an alumina crucible and kept in a preheated furnace at 600 °C for 10 min. Thus, a fluffy white product was obtained. The as-obtained powder was crushed to get a fine powder which was further washed twice with ethanol and deionised water and then put in the furnace again for the calcination at 1000 °C for 6 h. As received calcined samples (CYO:Ho^3+^,xEu^3+^) were used for further studies and analysis.

### Characterizations

The crystal structure and phase identification were studied by employing X-rays powder diffraction (XRD) using XRD-Rigaku Mini Flax 600 diffractometer with CuK_α_ radiation (λ = 1.54046 Å). The data were recorded in 2θ range 10° to 80° with a step size 0.02º/min. The TEM image was taken by Transmission Electron Microscope (TECHNAI G^2^ 20), operated at an accelerating voltage of 200 kV. To get an insight into the functional groups present in the as-synthesised samples, FTIR was employed using BRUKER-ALPHA II FT-IR spectrometer. The Raman vibrations were studied using Renishaw INVIA Raman Microscope with a 532 nm Nd-YAG laser excitation in 50–3500 cm^−1^ wavenumber range. UV–vis absorption spectra in diffuse reflectance mode were obtained using the UV–VIS-NIR Epoch 2 microplate reader Biotech, USA. The emission and excitation spectra of powder samples were recorded through a Photoluminescence spectrophotometer (Perkin Elmer, USA) and decay lifetime measurements were obtained using Fluorolog-3 (Horiba Jobin Yvon) spectrofluorometer excited with PMT attached 25 W pulsed Xenon lamp. All the measurements were performed at room temperature.
